# Development of the Food Acceptance Questionnaire for Thai Partial and Complete Edentulism

**DOI:** 10.3390/nu16101432

**Published:** 2024-05-09

**Authors:** Ketsupha Suwanarpa, Yoko Hasegawa, Jarin Paphangkorakit, Waranuch Pitiphat, Kazuhiro Hori, Takahiro Ono

**Affiliations:** 1 Department of Prosthodontics, Faculty of Dentistry, Khon Kaen University, Khon Kaen 40002, Thailand; ketssu@kku.ac.th; 2 Division of Comprehensive Prosthodontics, Faculty of Dentistry & Graduate School of Medical and Dental Sciences, Niigata University, 2-5274 Gakkocho-dori, Niigata 951-850014, Japan; hori@dent.niigata-u.ac.jp; 3 Department of Oral Biomedical Science, Faculty of Dentistry, Khon Kaen University, Khon Kaen 40002, Thailand; jarin@kku.ac.th; 4 Department of Preventive Dentistry, Faculty of Dentistry, Khon Kaen University, Khon Kaen 40002, Thailand; waranuch@kku.ac.th; 5 Department of Geriatric Dentistry, Osaka Dental University, 1-5-17 Otemae, Osaka 540-0008, Japan; ono-t@cc.osaka-dent.ac.jp

**Keywords:** aging, elderly, food, mastication, masticatory performance, questionnaires

## Abstract

This study aimed to develop the Food Acceptance Questionnaire (FAQ) to assess the masticatory ability of Thai older adults (≥60 years). Fifty participants were interviewed using open-ended questions about food they regularly consumed and avoided due to difficulty chewing. From a list of 140 items, 100 were recruited for a trial version of the FAQ. A total of 154 participants responded to the 5-point Likert scale on their chewing perception of each food item (1, impossible to chew; 5, most easily eaten). The average response of each food item was used as the chewing index (CI). The 100 food items were ranked and divided into five grades based on their CIs. Masticatory performance (MP) was objectively assessed by a visual scoring method using gummy jelly (UHA Mikakuto). Two foods from each grade that demonstrated the highest correlation with MP were selected to form the final 10-item FAQ. The FAQ score was calculated by summarizing the responses of 10 items. MP correlated strongly with the FAQ score (r = 0.57, *p* < 0.001), indicating its predictive validity. Furthermore, the FAQ indicates strong internal consistency (Cronbach’s alpha coefficient = 0.90), indicating high reliability. In conclusion, this newly developed 10-item FAQ is valid and reliable for assessing the masticatory ability of Thai older adults.

## 1. Background

The older population in most countries worldwide, including Thailand, has been increasing in recent years; as a result, Thailand has become an aged society [1]. Tooth loss is common among older adults, resulting in decreased masticatory ability and increased risk of systemic diseases, frailty, and mortality [2]. Masticatory performance (MP) is an essential indicator for assessing masticatory ability [3]. Most of the international research on MP to date has focused on the development of test methods and test materials for objective masticatory performance, such as sieve tests [4,5], hydrocolloid impression materials [6], paraffin wax cubes [7], optics [8], color-changeable chewing gum [9,10], and gummy jelly [11,12]. Objective assessments of the MP are believed to be more accurate than subjective assessments [13]. On the other hand, objective assessment requires special equipment and costs [14] and may be difficult to obtain in rural areas and underdeveloped countries.

The use of the Food Acceptance Questionnaire (FAQ), one of the methods used to assess subjective masticatory ability, can eliminate the limitations of objective assessment and may thus be a more convenient approach. Large population-based studies often employ FAQs due to their simplicity and low expenses [15,16]. The subjective masticatory ability assessment in response to the FAQ can be obtained through self-monitoring. Previous studies have used questionnaires to evaluate the difficulty of masticating ordinary foods, such as peanuts, raw carrots, apples, salami, bean curd, hard rice crackers, and steaks, and to determine food intake ability [16,17,18,19,20,21,22]. Hirai et al. reported that the correlation between the results of the gold-standardized masticatory performance test using sieve analysis [23] and Sato’s FAQ in patients with complete dentures showed a strong correlation between the chewing function score and chewing satisfaction [21], indicating that the FAQs were useful for evaluating masticatory ability. Shiga et al. reported a strong correlation between masticatory scores obtained from two types of FAQs and masticatory performance measured by the amount of glucose extracted during gummy jelly chewing [24]. Nevertheless, the FAQs used in these reports were limited to Japanese complete denture wearers, and the foods listed in these FAQs were Japanese foods [21,23]. FAQs were also invented by other nations, such as Korea [19], China [25], Canada [18,20], and the USA [22], and their common national foods influenced each questionnaire’s food lists. In Thailand, the use of the FAQ for assessing masticatory ability has been reported [26]; however, the characteristics of the FAQ are restricted to complete denture wearers and are not limited to older adults, and a questionnaire with high reliability and validity has not yet been obtained. In addition, the details of the FAQ development method are currently limited, and some studies reported the developing protocol but it might not be suitable for nationwide application [21,23,25].

Therefore, we focused on developing the FAQ for assessing the masticatory ability of Thai older adults with tooth loss. Because a food questionnaire with high predictive validity and reliability was needed, masticatory performance was determined by objective assessments using the gummy jelly test, and the results were compared for verification of the FAQ. In addition, we look forward to sharing our methodology as a prototype for developing FAQs in other regions.

## 2. Methods

The study protocol was approved by the Center for Ethics in Human Research of Khon Kaen University (#HE640294). Finite population correction coefficients were used to determine the sample size for creating the FAQ [27].

Sample size = N × [Z^2^ × p × (1 − p)/e^2^]/[N − 1 + (Z^2^ × p × (1 − p)/e^2^]

Assuming a population size of infinity, a confidence level of 95%, a margin of error of ±10%, and a response ratio of 0.5, the required sample size was 96. Assuming a dropout rate of 10%, a sample size of more than 106 persons was secured.

### 2.1. Participants

The participants were Thai men and women over 60 years old with partial dentate or complete edentulism who visited Khon Kaen University Dental Hospital from January 2020 to January 2022 for prosthodontic treatment. The inclusion criteria for subjects were missing one or more teeth (excluding third molars), no pain or severe inflammation in their teeth or periodontal tissue, and no problems with their dentures if they were wearing any dentures. The exclusion criteria were orofacial pain and significant cognitive or communication problems that may affect the ability to complete the questionnaire. The study objectives and procedures were explained. Those who met the inclusion criteria and were willing to provide written consent were enrolled. After exclusion, 154 participants exceeded the target sample size (75 men and 79 women).

The examiner conducted oral examinations with the participant lying on a dental chair in a supine position under sufficiently bright artificial lighting. The number of remaining teeth was assessed. The remaining teeth were defined as natural and treated teeth presenting in the oral cavity, including pontics and implants but excluding wisdom teeth, were impacted or had a high degree of torsion or slant.

### 2.2. FAQ Development

**Figure 1:**
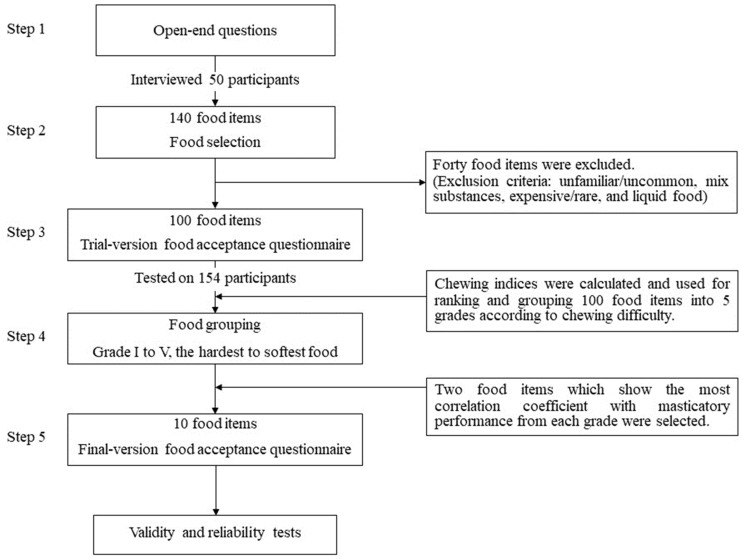
Flow diagram of food acceptance questionnaire development.

The following 5 steps were used for FAQ development (Figure 1):

Step 1: Open-ended questions

The examiner asked 50 participants open-ended questions by interviewing them about their current food choices, such as “What is the food you cannot eat or avoid due to chewing difficulty?” and “What food can you normally eat in your daily life?”

Step 2: Food selection

All the food items were collected from the interviews of the previous step of the open-ended questions. We considered that some food items should be excluded. There are four criteria for food exclusion: unfamiliar/uncommon food, mixed food, expensive/rare food, and liquid food.

Step 3: Trial version FAQ

After eliminating the excluded food items, all remaining food items were used to create the trial version FAQ. The trial version FAQ contained all remaining food items, and the responses on each food item were made on a 5-point Likert scale according to chewing difficulty from scores 1 to 5 (“impossible to chew” to “most easily eaten”). If the participant’s opinion was “do not eat because of dislike” or “never eaten”, the response was “0”. The color photographs of all food items were attached to the questionnaire to standardize the interpretation of the size, texture, and cooking process. All participants were asked to assign a mark on the coded response according to their perception of chewing difficulty.

Step 4: Food Grouping

We calculated the chewing index (CI) of each food by averaging the frequency of the coded responses. If any response was “0”, it was excluded from the CI calculation. The foods were then arranged in ascending order from the lowest to the highest CI. After that, the food items were shared equally into 5 grades (grades I to V: the hardest to the softest food). This grade was determined following the method of Sato et al. [21].

Step 5: Final version FAQ

The correlation (Pearson’s correlation coefficient) between the responses and the MP of each food was calculated. We chose two food items from each grade that showed the highest correlation coefficients with the MP. Finally, ten food items were included in the final version of the FAQ. If those food items were 5% or more, a response of 0 (do not eat because of dislike or never eaten) was eliminated to avoid further problems with incomplete answers or misinterpretation. To calculate the food acceptance score (FAQ), all responses to ten food items from the FAQ were collected. The maximum possible FAQ is 50, and the minimum FAQ is 10.

### 2.3. MP Assessments

The participants were instructed to chew a piece of gummy jelly (UHA Mikakuto Co., Ltd., Osaka, Japan) freely 30 times and, after chewing, to expectorate all the chewed fragments onto a piece of gauze spread over a paper cup. The collected pieces of gummy jelly were wrapped and washed with running tap water, and the gauze was stretched on a paper cup. The pieces of gummy jelly were spread out to prevent overlap of the chewed particles and evaluated by the visual scoring method, which categorizes the pieces into ten levels (0–9). The examiner decided on the score by comparing it with the visual scoring sheet [28]. The participants who regularly wore removable dentures were instructed to keep their dentures in their mouths during the MP assessment.

### 2.4. Statistical Analyses

The normality of the data distribution was examined using the Kolmogorov‒Smirnov test. When the data were non-normally distributed, a square root or logarithmic transformation was performed.

Age, number of remaining teeth, and posterior support were divided into three groups, which were then compared via analysis of variance (ANOVA) and multiple comparisons (Tukey’s test) to assess the characteristics of the participants.

For predictive validity assessment, we investigated the correlation of the FAQ with the MP (Pearson’s correlation coefficient). For reliability assessment, internal consistency analyses (corrected item-total correlation and Cronbach’s alpha) were used.

All analyses were performed using the SPSS software program, version 25.0, for Windows (IBM Corporation, Armonk, NY, USA), and probability values < 5% were considered significant.

## 3. Results

### 3.1. Baseline Participant Characteristics

The detailed characteristics of the participants and the relationships between masticatory ability (MP and FAQ score) and confounding factors are shown in Table 1. There were no significant sex differences in the MP/FAQ score ratio. In terms of age, the MP/FAQ score tended to increase as age decreased, and there were statistically significant differences in the MP between age groups. There was no significant difference in the FAQ score between age groups. The number of remaining teeth ranged from 0 to 27 (average: 16.5 teeth). There were statistically significant differences in the MP/FAQ between the group with more than 20 teeth and the other two groups. In terms of posterior support, there were statistically significant differences in the MP/FAQ between the posterior support group and the nonposterior support/edentulous group. There was no significant difference between the without posterior support group and the edentulous group.

### 3.2. FAQ Development

Step 1: Open-ended questions

One hundred forty food items were obtained from the interviews with the open-ended questionnaire (Appendix A, Table A1).

Step 2 and Step 3: Food selection and trial version of the FAQ

Forty food items were excluded, and one hundred food items remained and were included in the trial version of the FAQ.

Step 4: Food grouping

The distributions of the responses and the CI of each food item are shown in Appendix A, Table A2. One hundred food items were equally divided into five grades. Twenty food items were included in each grade.

Step 5: Final version FAQ

**Figure 2:**
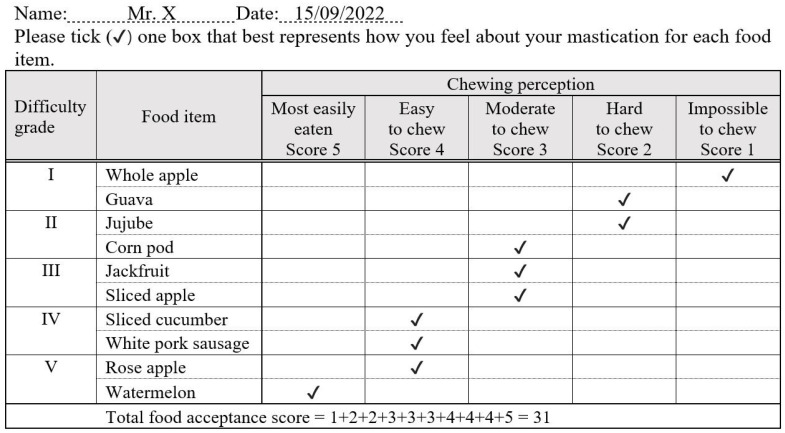
Final version Food Acceptance Questionnaire: an example of an answer to the questionnaire and food acceptance score calculation.

The correlation coefficients (Pearson’s correlation coefficients) between the responses and the MP of each food are also shown in Appendix A, Table A2. Two foods from each grade that showed the most correlation coefficients with the MP were selected. Ten food items were included in the final version of the FAQ, and the final version of the FAQ was represented (Figure 2).

### 3.3. Statistical Analyses

**Figure 3:**
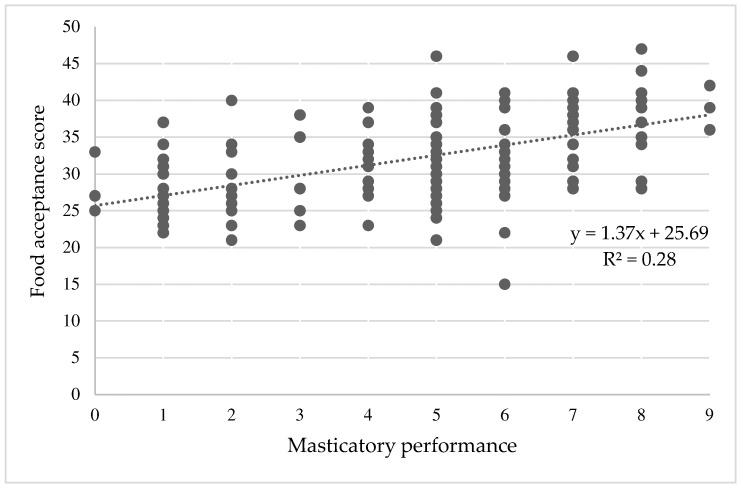
Relationship between food acceptance score and masticatory performance: the scatterplot with a positive correlation y, the value of the food acceptance score; x, the value of masticatory performance; R^2^, the coefficient of determination.

The relationship between the FAQ score and the MP score is presented in Figure 3. There was a significant correlation between the FAQ score and MP (r = 0.57, *p* < 0.001), indicating that participants with a higher FAQ score had greater MP.

The internal consistency analysis of the 10 food items demonstrated excellent consistency (Cronbach’s alpha = 0.90), and the corrected item-total correlation and Cronbach’s alpha for each food item deleted are shown in Table 2.

## 4. Discussion

It is generally agreed that an objective masticatory ability assessment is more accurate than a subjective assessment by the FAQ. However, using FAQ provides many benefits, such as convenience and low cost. Thus, the present study aimed to develop the validated FAQ as an alternative device for assessing masticatory ability, and we hope that the FAQ can be used as a guideline for FAQ development in other regions. Moreover, we preferred to cover all older adults with a wide range of masticatory functions, from very poor to superb. Our findings are supported by the wide variation in the MP of the study participants, as reflected by the even distribution of the visual score of gummy jelly.

This study selected gummy jelly as the standardized material for validating the FAQ and MP assessment because it has several advantages. The gummy jelly test has been proven to provide good measurement accuracy and discrimination ability [29]. We used the visual scoring method because this method is convenient and has sufficient validity [30].

In addition to FAQ development, which is the primary aim of this study, we also investigated the effect of some confounding factors on the MP/FAQ because we wanted to observe the tendency of the MP/FAQ to respond to such factors and the correspondence between the MP and FAQ. The results revealed that the MP/FAQ tended to decrease as age decreased and increased as the number of remaining teeth increased. Regarding posterior support, the MP/FAQ of the posterior support group was greater than that of the nonposterior support group and the edentulous group. These results coincided with those of previous studies [31,32,33,34,35]. Thus, we assumed that the developed FAQ could be a proper predictor of MP.

We adapted the food item grouping method from the original FAQ of Sato et al. [21]. The food items on the FAQ were categorized into five grades according to masticatory difficulty. However, our study included 10 food items, while Sato’s study included 20 food items. In addition, the difference from Satos’ questionnaire is the score calculation method. The FAQ in this study was calculated by summarizing the coded responses from the 5-point Likert scale selection of each food item, which can reflect more details of the scoring than Sato’s method. Sato’s FAQ asked the participants to describe the difficulty of eating, as follows: O, easy to chew; Δ, difficult to chew; or Χ, impossible to chew. To calculate the chewing function score, the total numbers of O, easy-to-chew responses were summed and presented as percentages. This study proved that 10 food items in the FAQ were sufficient for predicting MP, and the calculation method of the FAQ was validated by revealing a strong significant correlation between the FAQ and MP.

As mentioned above, we decided to categorize food into five grades according to chewing difficulty, from Grade I to Grade V, resembling the original FAQ of Sato et al. [21]. We considered the advantages of categorizing different food hardnesses and textures from the softest to the hardest food for the FAQ of Sato et al. As a result, the FAQ can reflect individual masticatory ability from the lowest to the highest. However, we noticed that the softer food items in Grade IV and Grade V, i.e., white pork sausage, rose apple, and watermelon, demonstrated low correlation coefficients to the MP (r = 0.37, 0.35, and 0.39, respectively). Thus, including these rather soft food items in the FAQ seemed worthless for discriminating the MP of an individual because nearly everyone can easily masticate. These soft food items might be excluded from the FAQ. In addition, the results of the internal consistency analyses of reliability supported the assumption that if watermelon was eliminated, the internal consistency remained excellent (Cronbach’s alpha if item deleted = 0.90). Nevertheless, we strongly considered including these soft food items because they are useful for patients with low masticatory ability and can help to provide a score for discrimination. Comparing the FAQ to the MP, including these soft food items, could help to cover all the information of patients who have low masticatory ability.

Similar to the original FAQ of Sato et al., there was no weighting of the difficulty of chewing food when calculating the FAQ. The difficulty of chewing food items was assigned the same weight as that of soft foods. In contrast, Hirai et al. presented their calculation method in which difficult-to-chew food has a larger coefficient weight, and the individual score is greater if more difficult-to-chew food can be chewed [23]. However, the calculation method of Hirai et al. seems to be complicated. Therefore, we decided to assign all food items to the same weight.

Regarding the participants’ characteristics, the participants in our study included both partially dentate and edentulous patients, while the participants in the study by Sato et al. had complete edentulism. In our study, 82% of participants were partially dentate and tended to have better chewing ability than did the edentulous participants in the study of Sato et al. However, we aimed to develop the FAQ for wide usage in even partially dentate or edentulous patients. Therefore, this instrument can provide an opportunity for evaluating perceived chewing difficulty in patients with minimal to complete tooth loss.

There are certain limitations in the present study that should be noted. Food acceptance and masticatory function, which are components of oral function, are influenced by various factors, such as the number of functional teeth, oral hygiene, occlusal force, salivary secretion, tongue-lip motor skill, swallowing function, experience with dentures, and stability and retention of dentures [3,36,37]. Additionally, older individuals with dentures often choose to exclude hard-to-chew foods from their daily diets [22,38]. However, none of these factors were taken into account in the analysis conducted in this study. Furthermore, because participants who did not completely answer all 10 food items were excluded from the calculation of the FAQ, the validity of the FAQ cannot be applied in the case of incomplete answers. Our study focused on this limitation if those food items with more than 5% dislike responses were not included in the FAQ. In addition, the FAQ developed in this study may not be suitable for populations who have limited food choices or vegetarianism. Some food items were Thai food; thus, this questionnaire cannot be used with other nations. A certain number of Thai people are Muslim and pork consumption is prohibited; as a result, they cannot rate the chewing difficulty of some food items, i.e., white pork sausage, and hence are unable to calculate the FAQ.

We considered designing a methodology for developing a FAQ that can be generalized and used in Thai older adults. One limitation was that most of the participants’ hometowns were located in the northeastern part of Thailand, which might have affected the inclusion of food items. The food items from the interviews tended to be local food. However, we eliminated this concern by setting the inclusion criteria for food selection for the trial version of the FAQ; thus, local food items were eliminated. However, we expect to prove the validity and test–retest reliability of the FAQ in other regional Thai populations for further study. The FAQ developed in the present study may not be widely applicable for assessing masticatory function, as it was tailored to Thai older adults at the University Dental Hospital and some foods included in the final version FAQ are Thai food. Indeed, we hope that this study can serve as a model for further developing FAQs in other regions.

## 5. Conclusions

This study presented the Food Acceptance Questionnaire development methodology and developed the 10-item FAQ for subjective masticatory function assessment in Thai older partial and complete edentulous patients. We found that the Food Acceptance Questionnaire is valid and reliable and can be used as an effective device to predict MP.

## Figures and Tables

**Table 1:** Participant characteristics and relationships between the masticatory performance/food acceptance score and other confounding factors.

	n (%)	Masticatory Performance		Food Acceptance Questionnaire Score	
		Mean ± SD	*p* Value	Mean ± SD	*p* Value
Gender					
All	154 (100)	4.8 ± 2.4	0.24	32.3 ± 6.1	0.38
Male	75 (48.7)	4.7 ± 2.3		32.5 ± 6.3	
Female	79 (51.3)	4.8 ± 2.5		32.1 ± 6.1	
Age					
60–69 years	103 (66.9)	5.3 ± 2.3	<0.001 ^a,b,c^	33.0 ± 6.3	0.18
70–79 years	45 (29.2)	4.3 ± 2.3		31.1 ± 5.8	
over 80 years	6 (3.9)	1.7 ± 1.6		30.8 ± 3.6	
Number of remaining teeth					
0–10 teeth	45 (29.2)	3.1 ± 2.1	<0.001 ^b,c^	29.2 ± 5.6	<0.001 ^b,c^
11–20 teeth	30 (19.5)	3.7 ± 2.1		29.9 ± 4.6	
Over 20 teeth	79 (51.3)	6.2 ± 1.7		35.1 ± 5.8	
Posterior support					
Posterior support	98 (63.6)	5.8 ± 1.9	<0.001 ^a,b^	32.3 ± 4.7	<0.001 ^a,b^
Nonposterior support	28 (18.2)	3.4 ± 2.1		27.0 ± 5.8	
Edentulous	28 (18.2)	2.6 ± 1.9		28.3 ± 3.9	

Note: SD: standard deviation. ^a^ Significant difference between 60 and 69 years of age and 70 and 79 years of age and between patients with posterior support and those with nonposterior support. ^b^ Significant difference between 60 and 69 years and over 80 years, between 0 and 10 teeth and over 20 teeth, and between patients with posterior support and edentulous patients. ^c^ Significant difference between those 70 and 79 years old and those over 80 years old and between those 11 and 20 years old and those over 20 years old. (*p* < 0.05, Student’s *t* test or Tukey’s test).

**Table 2:** Internal consistency of the Food Acceptance Questionnaire.

Difficulty Grade	Food Item	Internal Consistency	
		Corrected Item-Total Correlation	Cronbach’s Alpha If Item Deleted
I	Whole apple	0.65	0.89
	Guava	0.70	0.89
II	Jujube	0.76	0.89
	Corn pod	0.66	0.89
III	Jackfruit	0.68	0.89
	Sliced apple	0.71	0.89
IV	Sliced cucumber	0.70	089
	White pork sausage	0.62	0.90
V	Rose apple	0.62	0.90
	Watermelon	0.48	0.90

## Data Availability

The material described in the findings of this study, including all relevant raw data, will be freely available to any scientist wishing to use them for non-commercial purposes by contacting the corresponding author without breaching patient confidentiality.

## Appendix A

**Table A1:** 140 food items from the interview of open-ended questions.

No.	Food Item	Frequency of the Answers	
		Normally Eaten	Cannot Eaten or Avoided
1	Cooked rice	38	0
2	Sticky rice	22	5
3	Rice congee	18	0
4	Sliced raw papaya	12	3
5	Rice noodle	12	0
6	Boiled egg	12	0
7	Boiled pork	10	2
8	Fried chicken	10	1
9	Ripe papaya	10	0
10	Guava	8	5
11	Grilled pork	8	1
12	Grilled chicken	8	0
13	Banana	8	0
14	Egg bean curd	8	0
15	Tangerine	8	0
16	Stir-fried morning glory	6	1
17	Watermelon	6	0
18	Streamed fish	6	0
19	Grilled pork ball	5	1
20	Boiled chicken	5	0
21	Ripe mango	5	0
22	Slice of bread	5	0
23	Fried fish	5	0
24	Durian ^a^	5	0
25	Sliced apple	4	0
26	Potato chip	4	0
27	Boiled shrimp	4	0
28	Grape	4	0
29	Chinese cabbage	4	0
30	Fish ball	3	5
31	Peanut	3	5
32	Cantaloupe	3	2
33	Sliced cucumber	3	1
34	Rambutan	3	1
35	Minced pork	3	0
36	Stir-fried cabbage	3	0
37	Crispy bread butter	3	0
38	Boiled radish	3	0
39	Boiled straw mushroom	3	0
40	Rose apple	3	0
41	Jack fruit	3	0
42	Larb ^b^	3	0
43	Crispy pork	2	5
44	Unripe mango	2	2
45	Pork jerky	2	2
46	Stir-fried water mimosa	2	2
47	Bitter bean ^a^	2	2
48	Crispy fish	2	1
49	Chinese sausage	2	1
50	Soya duck	2	1
51	Glutinous rice balls	2	1
52	Poached mussels	2	1
53	Crisp rice cake	2	0
54	Pineapple	2	0
55	Cookie	2	0
56	Cherry tomato	2	0
57	Baby corn	2	0
58	Lotus root	2	0
59	Chicken sausage	2	0
60	White pork sausage	2	0
61	Crispy rice noodle	2	0
62	Stir-fried pork liver	2	0
63	Popcorn	2	0
64	Yogurt ^d^	2	0
65	Spicy shrimp paste dip ^d^	2	0
66	Streamed stuff bun ^b^	2	0
67	Ice cream ^d^	2	0
68	Boiled squid	1	7
69	Stir-fried Chinese kale	1	5
70	Biscuit stick	1	2
71	Jujube	1	2
72	Thai sweet crispy rice cake	1	2
73	Beef steak ^a^	1	2
74	Patongko	1	1
75	Dried mango paste	1	1
76	Boiled spotted babylon	1	1
77	Fried fish paste ball	1	1
78	Grilled banana	1	1
79	Winged bean	1	1
80	Fried taro	1	1
81	Mexican turnip	1	1
82	Sliced ginger	1	1
83	Date plum ^c^	1	1
84	Vietnamese noodles ^b^	1	0
85	Avocado ^a^	1	0
86	Raw fish (sashimi) ^a^	1	0
87	Spaghetti ^b^	1	0
88	Pie ^b^	1	0
89	Kimchi ^a^	1	0
90	Chocolate ^a^	1	0
91	Raw oyster ^a^	1	0
92	Fried spring rolls ^b^	1	0
93	Fermented pork sausage ^a^	1	0
94	Sandwich ^b^	1	0
95	Croissant ^a^	1	0
96	Grilled catfish ^a^	1	0
97	Crisp catfish flake salad ^b^	1	0
98	Sticky rice dumpling ^b^	1	0
99	Pizza ^b^	1	0
100	Northern Thai sausage ^a^	1	0
101	Pad Thai ^b^	1	0
102	Fried rice ^b^	1	0
103	Spicy horseshoe crab egg salad ^c^	1	0
104	Cheese ^a^	1	0
105	Cherry ^c^	1	0
106	Spicy stir-fried eel ^a^	1	0
107	Pork steak	0	5
108	Whole apple	0	5
109	Pork crackling	0	4
110	Pork cartilage	0	4
111	Round eggplant	0	4
112	Corn pod	0	4
113	Hard pork crackling	0	4
114	Sugarcane	0	4
115	Cow pea	0	3
116	Dried squid	0	3
117	Grilled pork intestine	0	3
118	Fried chicken tendon	0	3
119	Sweet cereal bar	0	3
120	Raw cabbage	0	2
121	Raw carrot	0	2
122	Chinese year cake	0	2
123	Cashew nut	0	2
124	Thai caramel toffee	0	2
125	Water chestnut	0	2
126	Coconut shoots	0	2
127	Thai caramelized crisp	0	2
128	Streamed crab ^c^	0	2
129	Ice ^d^	0	2
130	Roasted almond ^a^	0	2
131	Boiled shiitake mushroom	0	1
132	Gum	0	1
133	Banana chips	0	1
134	Okra	0	1
135	Chewy fish snack	0	1
136	Fried insects ^a^	0	1
137	Savory leaf wraps ^b^	0	1
138	Tapioka balls with pork filling ^b^	0	1
139	Baguette ^a^	0	1
140	Barbecued suckling pig ^c^	0	1

^a^ Foods were excluded due to unfamiliar or uncommon foods. ^b^ Foods were excluded due to mixed substances. ^c^ Foods were excluded due to expensive or rare foods. ^d^ Foods were excluded due to liquid food.

**Table A2:** One hundred food items were classified into 5 grades of masticatory difficulty, the participants’ responses to the trial version Food Acceptance Questionnaire were distributed, the chewing index of each food was calculated, and the correlation coefficient with masticatory performance was calculated.

Difficulty Grade	No.	Food Item	Frequency of the Chewing Perception (%)						Chewing Index ± SD	r
			Impossible to Chew Score 1	Hard to Chew Score 2	Moderate to Chew Score 3	Easy to Chew Score 4	Most Easily Eaten Score 5	Dislike/ Never Eaten Score 0		
I	1	Dried squid	83 (54)	43 (28)	17 (11)	4 (3)	0	7 (5)	1.6 ± 0.8	0.27 **
	2	Sugarcane	71 (46)	32 (21)	29 (19)	8 (5)	1 (1)	13 (8)	1.8 ± 1.0	0.36 **
	3	Grilled pork intestine	41 (27)	70 (46)	29 (19)	9 (6)	1 (1)	4 (3)	2.1 ± 0.9	0.24 **
	4	Hard pork crackling	46 (30)	55 (36)	33 (21)	12 (8)	3 (2)	5 (3)	2.1 ± 1.0	0.18 *
	5	Chicken tendon	47 (31)	48 (31)	34 (22)	9 (6)	1 (1)	15 (10)	2.1 ± 1.0	0.37 **
	6	Whole apple ^a^	39 (25)	54 (35)	42 (27)	9 (6)	3 (2)	7 (5)	2.2 ± 1.0	0.49 **
	7	Boiled squid	29 (19)	54 (35)	52 (34)	12 (8)	7 (5)	0	2.3 ± 0.9	0.34 **
	8	Pork cartilage	34 (22)	48 (31)	57 (37)	12 (8)	0	3 (2)	2.3 ± 0.9	0.18 *
	9	Thai caramelized crisp	31 (20)	59 (38)	35 (23)	21 (14)	1 (1)	7 (5)	2.3 ± 1.0	0.40 **
	10	Sweet cereal bar	32 (21)	52 (34)	44 (29)	18 (12)	3 (2)	5 (3)	2.4 ± 1.0	0.38 **
	11	Pork jerky	21 (14)	45 (29)	69 (45)	15 (10)	1 (1)	3 (2)	2.5 ± 0.9	0.24 **
	12	Crispy pork	20 (13)	56 (36)	52 (34)	20 (13)	1 (1)	5 (3)	2.5 ± 0.9	0.34 **
	13	Pork crackling	24 (16)	51 (33)	49 (32)	14 (9)	4 (3)	12 (8)	2.5 ± 1.0	0.12
	14	Raw carrot	19 (12)	37 (24)	65 (42)	19 (12)	2 (1)	12 (8)	2.6 ± 0.9	0.45 **
	15	Stir-fried water mimosa	18 (12)	56 (36)	48 (31)	22 (14)	3 (2)	7 (5)	2.6 ± 1.0	0.38 **
	16	Pork steak	9 (6)	46 (30)	58 (38)	24 (16)	2 (1)	15 (10)	2.7 ± 0.9	0.33 **
	17	Unripe mango	11 (7)	47 (31)	68 (44)	24 (16)	2 (1)	2 (1)	2.7 ± 0.9	0.38 **
	18	Guava ^a^	15 (10)	55 (36)	49 (32)	31 (20)	3 (2)	1 (1)	2.7 ± 0.9	0.43 **
	19	Thai caramel toffee	16 (10)	45 (29)	59 (38)	29 (19)	0	5 (3)	2.7 ± 0.9	0.39 **
	20	Fried chicken	6 (4)	39 (25)	82 (53)	23 (15)	1 (1)	3 (2)	2.8 ± 0.8	0.16
II	21	Round eggplant	6 (4)	41 (27)	73 (48)	26 (17)	2 (1)	6 (4)	2.8 ± 0.8	0.42 **
	22	Crispy fish	9 (6)	45 (29)	67 (44)	30 (20)	1 (1)	2 (1)	2.8 ± 0.9	0.18 *
	23	Stir-fried Chinese kale	8 (5)	47 (31)	65 (42)	27 (18)	4 (3)	3 (2)	2.8 ± 0.9	0.38 **
	24	Boiled spotted babylon	8 (5)	39 (25)	48 (31)	22 (14)	4 (3)	33 (21)	2.8 ± 0.9	0.23 *
	25	Dried mango paste	18 (12)	43 (28)	53 (34)	32 (21)	3 (2)	5 (3)	2.8 ± 1.0	0.28 **
	26	Cow pea	12 (8)	29 (19)	74 (48)	33 (21)	4 (3)	2 (1)	2.9 ± 0.9	0.38 **
	27	Chinese year cake	5 (3)	48 (31)	59 (38)	34 (22)	2 (1)	6 (4)	2.9 ± 0.9	0.29 **
	28	Biscuit stick	7 (5)	39 (25)	58 (38)	38 (25)	0	12 (8)	2.9 ± 0.9	0.35 **
	29	Chewy fish snack	7 (5)	36 (23)	69 (45)	29 (19)	4 (3)	9 (6)	2.9 ± 0.9	0.31 **
	30	Peanut	12 (8)	41 (27)	62 (40)	32 (21)	6 (4)	1 (1)	2.9 ± 1.0	0.34 **
	31	Soya duck	0	43 (28)	69 (45)	35 (23)	0	7 (5)	3.0 ± 0.7	0.34 **
	32	Boiled pork	3 (2)	36 (23)	76 (49)	29 (19)	5 (3)	5 (3)	3.0 ± 0.8	0.29 **
	33	Grilled chicken	4 (3)	36 (23)	78 (51)	36 (23)	0	0	3.0 ± 0.8	0.29 **
	34	Sliced raw papaya	10 (7)	33 (21)	70 (46)	35 (23)	6 (4)	0	3.0 ± 0.9	0.38 **
	35	Stir-fried morning glory	6 (4)	42 (27)	62 (40)	35 (23)	8 (5)	1 (1)	3.0 ± 0.9	0.43 **
	36	Cashew nut	8 (5)	33 (21)	67 (44)	40 (26)	4 (3)	2 (1)	3.0 ± 0.9	0.39 **
	37	Banana chips	7 (5)	33 (21)	65 (42)	41 (27)	4 (23)	4 (3)	3.0 ± 0.9	0.23 **
	38	Jujube ^a^	6 (4)	39 (25)	65 (42)	39 (25)	4 (3)	1 (1)	3.0 ± 0.9	0.48 **
	39	Corn pod ^a^	12 (8)	30 (20)	66 (43)	39 (25)	6 (4)	1 (1)	3.0 ± 1.0	0.44 **
	40	Mexican turnip	6 (4)	41 (27)	55 (36)	38 (25)	8 (5)	6 (4)	3.0 ± 1.0	0.43 **
III	41	Water chestnut	8 (5)	36 (23)	57 (37)	42 (27)	8 (5)	3 (2)	3.0 ± 1.0	0.40 **
	42	Thai sweet crispy rice cake	12 (8)	35 (23)	59 (38)	43 (28)	4 (3)	1 (1)	3.0 ± 1.0	0.35 **
	43	Gum	14 (9)	34 (22)	30 (20)	35 (23)	11 (7)	30 (20)	3.0 ± 1.2	0.33 **
	44	Winged beans	6 (4)	18 (12)	77 (50)	39 (25)	6 (4)	8 (5)	3.1 ± 0.8	0.39 **
	45	Fried taro stick	3 (2)	27 (18)	73 (47)	40 (26)	7 (5)	4 (3)	3.1 ± 0.8	0.30 **
	46	Popcorn	3 (2)	29 (19)	69 (45)	45 (29)	6 (4)	2 (1)	3.1 ± 0.8	0.35 **
	47	Boiled shiitake mushroom	6 (4)	27 (18)	71 (46)	36 (23)	11 (7)	3 (2)	3.1 ± 0.9	0.20 *
	48	Raw cabbage	4 (3)	28 (18)	66 (43)	39 (25)	8 (5)	9 (6)	3.1 ± 0.9	0.43 **
	49	Poached mussels	5 (3)	28 (18)	65 (42)	42 (27)	6 (4)	8 (5)	3.1 ± 0.9	0.28 **
	50	Coconut shoots	3 (2)	31 (20)	65 (42)	41 (27)	8 (5)	6 (4)	3.1 ± 0.9	0.38 **
	51	Okra	8 (5)	28 (18)	51 (33)	36 (23)	7 (5)	24 (16)	3.1 ± 1.0	0.35 **
	52	Sliced ginger	6 (4)	37 (24)	48 (31)	44 (29)	6 (4)	13 (8)	3.1 ± 1.0	0.40 **
	53	Boiled chicken	2 (2)	21 (13)	77 (50)	47 (31)	4 (3)	3 (2)	3.2 ± 0.8	0.32 **
	54	Chinese sausage	3 (2)	20 (13)	75 (49)	40 (26)	7 (5)	9 (6)	3.2 ± 0.8	0.19 *
	55	Grilled pork	1 (1)	23 (15)	75 (49)	46 (30)	5 (3)	4 (3)	3.2 ± 0.8	0.28 **
	56	Jackfruit ^a^	5 (3)	21 (14)	69 (45)	48 (31)	9 (6)	2 (1)	3.2 ± 0.9	0.48 **
	57	Sliced apple ^a^	1 (1)	12 (8)	84 (55)	47 (31)	10 (7)	0	3.3 ± 0.7	0.41 **
	58	Grilled pork ball	4 (3)	19 (12)	70 (46)	48 (31)	8 (5)	5 (3)	3.3 ± 0.8	0.39 **
	59	Stir-fried pork liver	2 (1)	17 (11)	69 (45)	39 (25)	9 (6)	18 (12)	3.3 ± 0.8	0.32 **
	60	Grilled banana	4 (3)	19 (12)	63 (41)	55 (36)	12 (8)	1 (1)	3.3 ± 0.9	0.34 **
IV	61	Sliced cucumber ^a^	0	16 (10)	67 (44)	60 (39)	11 (7)	0	3.4 ± 0.8	0.40 **
	62	Patongko	1 (1)	16 (10)	62 (40)	56 (36)	14 (9)	5 (3)	3.4 ± 0.8	0.21 *
	63	Lotus root	1 (1)	17 (11)	65 (43)	55 (36)	8 (5)	8 (5)	3.4 ± 0.8	0.26 **
	64	Chicken sausage	3 (2)	10 (7)	64 (42)	62 (40)	10 (7)	5 (3)	3.4 ± 0.8	0.29 **
	65	Fried fish paste ball	1 (1)	16 (10)	62 (40)	61 (40)	8 (5)	6 (4)	3.4 ± 0.8	0.30 **
	66	Fish ball	7 (5)	14 (9)	55 (36)	57 (37)	19 (12)	2 (1)	3.4 ± 1.0	0.35 **
	67	Sticky rice	1 (1)	6 (4)	68 (44)	67 (44)	12 (8)	0	3.5 ± 0.7	0.35 *
	68	White pork sausage ^a^	0	11 (7)	63 (41)	64 (42)	11 (7)	5 (3)	3.5 ± 0.7	0.37 **
	69	Stir-fried cabbage	2 (1)	14 (9)	57 (37)	64 (42)	13 (8)	4 (3)	3.5 ± 0.8	0.32 **
	70	Rambutan	2 (1)	16 (10)	57 (37)	66 (43)	13 (8)	0	3.5 ± 0.8	0.28 **
	71	Baby corn	2 (1)	7 (5)	73 (47)	57 (37)	13 (8)	2 (1)	3.5 ± 0.8	0.29 **
	72	Chinese cabbage	1 (1)	9 (6)	64 (42)	61 (40)	14 (9)	5 (3)	3.5 ± 0.8	0.35 **
	73	Crispy rice noodle	0	5 (3)	69 (45)	61 (40)	14 (9)	5 (3)	3.6 ± 0.7	0.35 **
	74	Fried fish	1 (1)	7 (5)	62 (40)	67 (44)	17 (11)	0	3.6 ± 0.8	0.24 **
	75	Boiled shrimp	0	7 (5)	40 (26)	93 (60)	14 (9)	0	3.7 ± 0.8	0.29 **
	76	Glutinous rice balls	2 (1)	11 (7)	41 (27)	70 (46)	23 (15)	7 (5)	3.7 ± 0.9	0.27 **
	77	Cookie	0	7 (5)	46 (30)	75 (48)	22 (14)	4 (3)	3.8 ± 0.7	0.30 **
	78	Crisp rice cake	1 (1)	5 (3)	51 (33)	69 (45)	26 (17)	2 (1)	3.8 ± 0.8	0.21 **
	79	Cherry tomato	0	9 (6)	36 (23)	77 (50)	28 (18)	4 (3)	3.8 ± 0.8	0.33 **
	80	Crispy bread butter	1 (1)	7 (5)	42 (27)	78 (51)	24 (16)	2 (1)	3.8 ± 0.8	0.28 **
V	81	Straw mushroom	2 (1)	6 (3.9)	42 (27)	76 (49)	28 (18)	0	3.8 ± 0.8	0.24 **
	82	Cantaloupe	0	7 (4.5)	42 (27)	75 (49)	26 (17)	4 (3)	3.8 ± 0.8	0.28 **
	83	Rose apple ^a^	2 (1)	4 (2.6)	33 (21)	91 (59)	23 (15)	1 (1)	3.8 ± 0.8	0.35 **
	84	Pineapple	0	2 (1.3)	44 (29)	75 (49)	32 (21)	1 (1)	3.9 ± 0.7	0.27 **
	85	Minced pork	0	3 (1.9)	37 (24)	88 (57)	22 (14)	4 (3)	3.9 ± 0.7	0.15
	86	Grape	1 (1)	3 (2)	29 (19)	91 (59)	29 (19)	1 (1)	3.9 ± 0.7	0.30 **
	87	Potato chip	0	7 (5)	40 (26)	69 (45)	33 (21)	5 (3)	3.9 ± 0.8	0.24 **
	88	Boiled radish	2 (1)	2 (1)	27 (18)	78 (51)	40 (26)	5 (3)	4.0 ± 0.8	0.30 **
	89	Tangerine	2 (1)	1 (1)	22 (14)	92 (60)	37 (24)	0	4.1 ± 0.7	0.20 *
	90	Slice of bread	0	1 (1)	28 (18)	80 (52)	42 (27)	3 (2)	4.1 ±0.7	0.30 **
	91	Rice noodle	0	0	8 (5)	94 (61)	52 (34)	0	4.3 ± 0.6	0.32 **
	92	Ripe mango	0	0	16 (10)	80 (52)	57 (37)	1 (1)	4.3 ± 0.6	0.26 **
	93	Cooked rice	0	2 (1)	12 (8)	86 (56)	54 (35)	0	4.3 ± 0.7	0.14
	94	Watermelon ^a^	0	0	5 (3)	75 (49)	73 (47)	1)	4.4 ± 0.6	0.39 **
	95	Streamed fish	0	0	11 (7)	67 (44)	74 (48)	2 (1)	4.4 ± 0.6	0.21 **
	96	Boiled egg	0	0	6 (4)	72 (47)	75 (49)	1 (1)	4.5 ± 0.6	0.17 *
	97	Banana	1 (1)	1 (1)	5 (3)	61 (40)	86 (56)	0	4.5 ± 0.7	0.24 **
	98	Ripe papaya	0	0	4 (3)	56 (36)	93 (60)	1 (1)	4.6 ± 0.5	0.26 **
	99	Eggs bean curd	0	0	5 (3)	36 (23)	110 (71)	3 (2)	4.7 ± 0.5	0.18 *
	100	Rice congee	0	0	0	5 (3)	148 (96)	1 (1)	5.0 ± 0.2	0.12 *

Note: SD, standard deviation; r, Pearson’s correlation coefficient between the responses and the masticatory performance; * *p* value < 0.05; ** *p* value < 0.01. ^a^ Foods were selected.
